# Chemical Tools for Targeted Amplification of Reactive Oxygen Species in Neutrophils

**DOI:** 10.3389/fimmu.2018.01827

**Published:** 2018-08-13

**Authors:** Viktor Reshetnikov, Jonas Hahn, Christian Maueröder, Christine Czegley, Luis Enrique Munoz, Martin Herrmann, Markus H. Hoffmann, Andriy Mokhir

**Affiliations:** ^1^Department of Chemistry and Pharmacy, Organic Chemistry II, Friedrich-Alexander-Universität Erlangen-Nürnberg, Erlangen, Germany; ^2^Department of Internal Medicine 3 – Rheumatology and Immunology, University Hospital Erlangen, Friedrich-Alexander-Universität Erlangen-Nürnberg, Erlangen, Germany; ^3^Cell Clearance in Health and Disease Lab, VIB Center for Inflammation Research, Ghent, Belgium; ^4^Department of Biomedical Molecular Biology, Ghent university, Ghent, Belgium

**Keywords:** aminoferrocenes, autoimmune disease, chronic granulomatous disease, inflammation, NADPH oxidase 2, neutrophils, reactive oxygen species, therapy

## Abstract

A number of chemical compounds are known, which amplify the availability of reactive oxygen species (ROS) in neutrophils both *in vitro* and *in vivo*. They can be roughly classified into NADPH oxidase 2 (NOX2)-dependent and NOX2-independent reagents. NOX2 activation is triggered by protein kinase C agonists (e.g., phorbol esters, transition metal ions), redox mediators (e.g., paraquat) or formyl peptide receptor (FPR) agonists (e.g., aromatic hydrazine derivatives). NOX2-independent mechanisms are realized by reagents affecting glutathione homeostasis (e.g., l-buthionine sulfoximine), modulators of the mitochondrial respiratory chain (e.g., ionophores, inositol mimics, and agonists of peroxisome proliferator-activated receptor γ) and chemical ROS amplifiers [e.g., aminoferrocene-based prodrugs (ABPs)]. Since a number of inflammatory and autoimmune diseases, as well as cancer and bacterial infections, are triggered or enhanced by aberrant ROS production in neutrophils, it is tempting to use ROS amplifiers as drugs for the treatment of these diseases. However, since the known reagents are not cell specific, their application for treatment likely causes systemic enhancement of oxidative stress, leading to severe side effects. Cell-targeted ROS enhancement can be achieved either by using conjugates of ROS amplifiers with ligands binding to receptors expressed on neutrophils (e.g., the GPI-anchored myeloid differentiation marker Ly6G or FPR) or by designing reagents activated by neutrophil function [e.g., phagocytic activity or enzymatic activity of neutrophil elastase (NE)]. Since binding of an artificial ligand to a receptor may trigger or inhibit priming of neutrophils the latter approach has a smaller potential for severe side effects and is probably better suitable for therapy. Here, we review current approaches for the use of ROS amplifiers and discuss their applicability for treatment. As an example, we suggest a possible design of neutrophil-specific ROS amplifiers, which are based on NE-activated ABPs.

## Introduction

**Figure 1:**
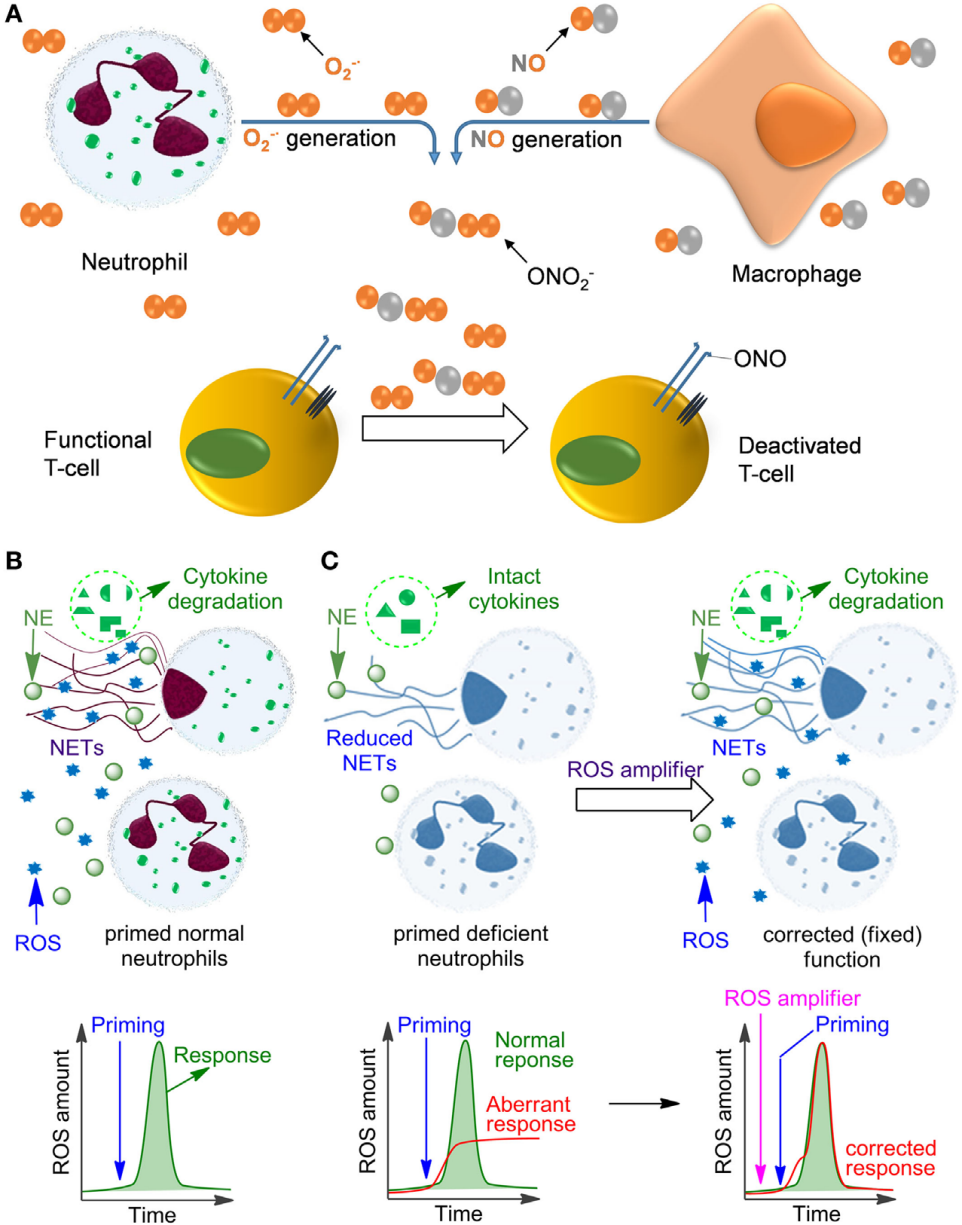
Influence of reactive oxygen species (ROS) on immune cell function. **(A)** The mechanism of deactivation of T-cells by ROS and reactive nitrogen species produced by neutrophils and monocytes/macrophages. This process is facilitated by the proximity of macrophages and T-cells at the sites of infection due to binding of the T-cells to antigens presented on the macrophage surface. **(B)** Oxidative burst in normal primed neutrophils leading to ROS production and neutrophil extracellular trap (NET) formation. **(C)** Aberrant response of NADPH oxidase 2-deficient neutrophils (e.g., in chronic granulomatous disease) leading to low ROS and insufficient NET formation. This deficiency can be fixed by applying ROS amplifiers.

Neutrophils are formed from stem cells in the bone marrow and constitute roughly 40–75% of the leukocyte population in humans, which makes them the most abundant white blood cells. They play a key role in the response of the innate immune system toward both infectious and sterile agents. These cells possess high mobility due to their characteristic segmented nuclei that coined them the name polymorphonuclear (PMN) cells. Therefore, neutrophils can quickly migrate from blood to the site of inflammation, where they respond with either phagocytosis of the inflammatory trigger or with degranulation, finally resulting in disintegration of pathogens. Alternatively, neutrophils release DNA-rich neutrophil extracellular traps (NETs), which entrap and neutralize infectious pathogens and sterile factors (1, 2). Additionally, neutrophils recruit macrophages, activate dendritic cells, trigger production of antibodies, and stimulate CD4^+^ and CD8^+^ T cells, thereby affecting the adaptive immune system (3–5). In many of these crucial processes the generation of reactive oxygen species (ROS) plays a key role, either because ROS act directly as cytotoxic agents against pathogens or as important regulators of inflammatory responses including, e.g., NET formation, secretion of various proteases, redox enzymes, and antimicrobial factors. In fully functional primed neutrophils, ROS are mainly produced by NADPH oxidase 2 (NOX2) resulting in the so-called oxidative burst. In contrast to its pro-inflammatory role during the early phase of the fight against infections, ROS can also inhibit inflammatory responses (6), e.g., by deactivation of T cells (7–10) or by degradation of inflammatory mediators in NETs (11–13) (Figure 1). In agreement with these functions, insufficient ROS production by neutrophils, e.g., due to NOX2 deficiency results in persistent infections but also in autoimmunity and non-resolving inflammation, as can be observed in chronic granulomatous disease (CGD) (14–17).

Despite modern clinical management, long-term outcomes in patients with CGD are still bleak, especially for those individuals with fewer than 10% neutrophils with normal oxidase activity (18, 19). Hematopoietic stem cell transplantations, as the first-line definitive therapy in CGD, are often hampered by limitations in available matched bone marrow donors and the risk of graft-versus-host disease (20). In the last years, technical advantages have also paved the way for specific gene editing to restore proteins encoded by genes carrying loss-of-function mutations [reviewed in Ref. (21)]. Particularly, gene therapy using lentiviral vectors enabling specific expression in myeloid cells (22) and approaches employing the clustered regularly interspaced short palindromic repeats/Cas9 system (23) have obtained promising results.

An alternative approach would be the use of drugs that are capable to trigger ROS generation in NOX2-deficient neutrophils. Such compounds would have the potential to both stimulate the immune system and prevent chronic inflammation (24). In this review, we summarize chemical substances that reportedly can be used for stimulation of ROS production in neutrophils. We also discuss mechanisms of action as well as problems in application of these compounds as drugs and suggest possible solutions.

## Formation of ROS Upon Priming of Functional Neutrophils and Their Mutual Transformations in Live Organisms

**Figure 2:**
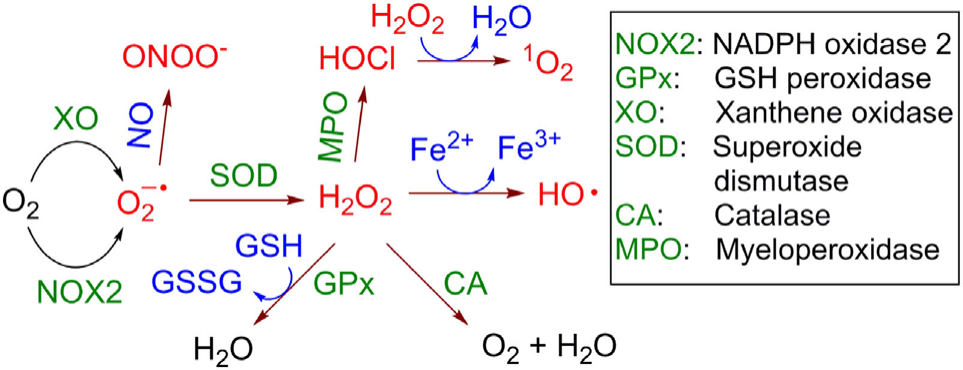
Generation and transformation of reactive oxygen species in live cells. Reactive species are red colored.

The multienzymatic NOX2 is activated upon neutrophil priming. In the active state it is able to catalyze one-electron reduction of molecular oxygen (^3^O_2_) with the formation of a superoxide anion radical $\left({\text{O}}_{2}^{-}•\right)$ (Figure 2). This reactive and, therefore, short-lived anion is one of the key precursors of other ROS in cells and the extracellular space, including hydrogen peroxide (H_2_O_2_), hydroxyl radicals (HO•), hypochlorous acid (HOCl) and its anion hypochlorite (ClO^−^), singlet oxygen (^1^O_2_), as well as a series of reactive nitrogen species, e.g., peroxynitrite (ONOO^−^) and carbon-centered (R•), alkoxy- (RO•) and alkylperoxy-radicals (ROO•). In aqueous solution ${\text{O}}_{2}^{-}•$ is dismutated spontaneously with formation of H_2_O_2_ and ^3^O_2_. This reaction is accelerated over 10^4^-fold in the presence of superoxide dismutase. In contrast to ${\text{O}}_{2}^{-}•$, H_2_O_2_ is a stable molecule. For example, concentrated aqueous solutions of H_2_O_2_ are commercially available, can be safely delivered over long distances and stored over extended time. Apart from NOX2, the protein folding machinery in the endoplasmic reticulum (ER) also causes generation of ROS (25).

H_2_O_2_ is able to cross the cellular membrane, e.g., *via* the aquaporin-mediated pathway (26, 27). Therefore, it is distributed over both the intracellular and extracellular space independently of its site of generation. Though H_2_O_2_ is itself not a toxic molecule, in the presence of electron donors, e.g., Cu^+^ or Fe^2+^, it is reduced with cleavage of the O-O bond leading to formation of the hydroxide anion HO^−^ and the HO• radical (Fenton reaction, Figure 2). HO• radicals are extremely reactive and, therefore, short-lived. They are capable of subtracting a hydrogen atom (“H”) even from very stable (bio)molecules, e.g., lipids, nucleic acids, proteins that leads to formation of a variety of organic radicals (e.g., R•, RO•, and ROO•), deactivation of the biomolecules, and ultimately induction of cell death *via* different pathways, e.g., apoptosis, necrosis, or the formation of NETs. Other reactions leading to H_2_O_2_ elimination in cells include catalase (CA)-induced conversion of H_2_O_2_ to water and molecular oxygen (^3^O_2_) and glutathione peroxidase-catalyzed reduction of H_2_O_2_ in the presence of glutathione (GSH) with the formation of glutathione disulfide (GSSG) and water. Interestingly, H_2_O_2_ is accumulated in some organelles, e.g., lysosomes (LY) and the ER. This can be explained by the low catalase activity in LY (28) and the low concentration of reduced GSH in ER relatively to its concentration in cytoplasm (29).

In neutrophils, H_2_O_2_ is also used as a substrate of myeloperoxidase (MPO), which transforms Cl^−^ anions to highly reactive HOCl. At pH 7 the latter acid (pK_a_~7.5) is partially dissociated, forming ClO^−^ anions. In the MPO-catalyzed reaction both H_2_O_2_ and ClO^−^ co-exist for some time in solution. At these conditions electronically excited form of molecular oxygen ^1^O_2_ is formed with high yield (30). In contrast to highly reactive HO• and ${\text{O}}_{2}^{-}•$, which act locally at the site of their generation, ^1^O_2_ exhibits extended lifetime in aqueous solution of ~3 μs and can migrate over 100 nm after its generation (31).

Furthermore, nitric oxide (NO•), which is a rather reactive inorganic radical, is generated from l-arginine in the presence of inducible NO-synthase in activated macrophages (32) and to the lesser degree in primed neutrophils themselves (33). Since macrophages are located at the infection site in close proximity to neutrophils, NO• produced by these cells can combine with ${\text{O}}_{2}^{-}•$ generated by neutrophils in an extremely quick reaction with the formation of highly reactive ONOO^−^. All these species (ROS, NO•, and ONOO^−^) chemically modify and in this way deactivate extracellular receptors of neighboring T cells or even cause their apoptosis and necrosis (8–10).

## Stimulation of ROS Production in Neutrophils by Chemical Compounds

Chemical and natural compounds amplifying the ROS amount in neutrophils can be classified as NOX2-dependent modulators, which are more common, and NOX2-independent ones.

### NOX2-Dependent ROS Modulators

#### Protein Kinase C (PKC) Agonists

**Figure 3:**
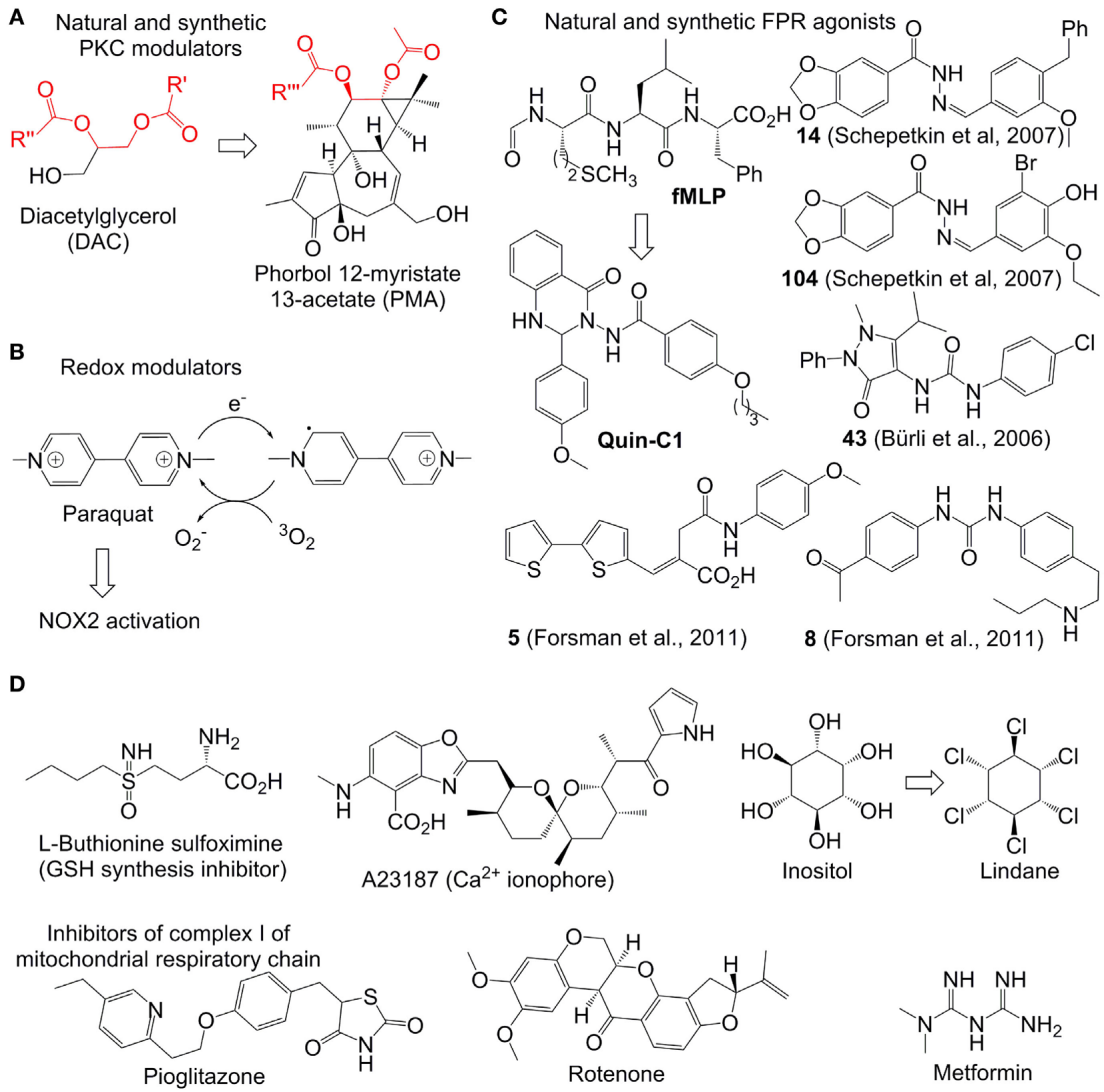
Representative reactive oxygen species (ROS) modulators in neutrophils. **(A–C)**: NADPH oxidase 2(NOX2)-dependent modulators. Quin-C1 is an formyl peptide receptor agonist, but does not induce ROS in cells. **(D)** NOX2-independent modulators. Structurally related fragments in DAC and phorbol myristate acetate are indicated with red color.

After its activation within cells, PKC catalyzes phosphorylation of the p47^phox^ subunit of the protein associate p47^phox^/SH3/p7^phox^ (Figure 3A). The resulting product migrates to the cellular membrane, where it is assembled due to the interaction between SH3 and p22^phox^ subunits with formation of the functional NOX2 system (34). Therefore, compounds enhancing the PKC activity (PKC agonists) are expected to stimulate NOX2 formation and correspondingly increase ROS generation. Examples of such agonists include a variety of hydrophobic phorbol esters, e.g., phorbol myristate acetate (PMA) and phorbol dibutyrate (PDB). These compounds act in this way due to their similarity to the natural activator of PKC, diacylglycerol (DAC) (Figure 3A). They are broadly used in immunological research. However, these esters exhibit a number of undesired side effects *in vivo*, which prevents their therapeutic applications. For example, PMA is oncogenic and can cause fever (35). The effects of phorbol esters are attenuated by diphenyleneiodonium (DPI), a commonly used unspecific covalent inhibitor of NOX2, which binds to reduced flavin adenine dinucleotide in the gp91^phox^ subunit (36). These data confirm that the increased ROS production in neutrophils in response PMA is derived from the NOX2 activity.

The Ca^2+^-ionophore A23187 (Figure 3D) was found to further enhance the ROS-production in PDB-treated neutrophils (37). This synergy is logical, since the increased amount of intracellular Ca^2+^, caused by the treatment with Ca^2+^-ionophores such as A23187 and ionomycin, is expected to further stimulate PKC. Additionally to that, it has been found that Ca^2+^ strengthens binding of phorbol esters to their receptors (37). A number of other natural and synthetic agonists of PKC, which are either analogs of phorbol esters or unrelated structures, have been reported. However, clinical application of all known agonists is limited by substantial side effects (38).

Fully inorganic PKC activators, which are known to increase ROS in neutrophils *in vitro*, include ionic salts of the soft transition metal ions Zn^2+^, Cd^2+^, and Ni^2+^ ions. They are usually only active at the high concentration of ≥1 mM (39, 40). The metal ion effect is strongly dependent on the presence of chelating agents in the medium. Otherwise, the mechanism of activation is not known.

#### Redox Mediators

Paraquat is an intensely colored, dicationic 4,4′-bipyridinium salt (other name: methyl viologen) (Figure 3B). This compound is used as a broad spectrum herbicide. Its mode of action relies on its powerful electron acceptor properties. For example, in plants it accepts an electron from photosystem I with formation of a resonance-stabilized organic radical (Figure 3B). The latter species transfer the electron further to molecular oxygen with formation of ${\text{O}}_{2}^{-}•$ followed by generation of other ROS (as described above). The toxic effects of ROS generation in plants explain the herbicidal properties of paraquat. In humans this drug is accumulated in lungs, where it catalyzes ROS production by mediation of the electron transfer to oxygen, analogously to its effect in plants, that leads to acute lung injury (41). In mammalian cells, NAPDH can potentially act as donor of electrons for paraquat (42). It has been reported that in neutrophils paraquat-induced ROS generation activates p38 MAPK and NF-kB signaling pathways thereby delaying neutrophil apoptosis. This effect is fully blocked by NOX2 inhibitors and partially blocked by PKC inhibitors confirming the involvement of the latter two enzymes in the paraquat-induced activation of neutrophils (43, 44).

#### Formyl Peptide Receptor (FPR) Agonists

Formyl peptide receptor is a G-protein coupled receptor expressed on the neutrophil membrane (45). Its stimulation by the bacteria-specific peptide formylmethionyl-leucyl-phenylalanine (fMLF) activates NOX2 thereby inducing ROS generation (Figure 3C). A number of organic molecules have been discovered, which act as fMLF mimics and, therefore, are able to stimulate ROS production in functional neutrophils. They include compounds 14, 104 (46), 43 (A) (47, 48), 5, and 10 (49). In contrast to the parent stimulant fMLF, all synthetic ligands are heterocyclic compounds. It is worth noting that many potent synthetic FPR ligands reported in the literature contain one N-N bond (Figure 3C). The role of this structural element still remains to be clarified.

Not all FPR agonists induce ROS production in neutrophils. One such example is the quinazolinone C derivative Quin-C1 (50, 51). Though it does not generate ROS, it induces mobilization of Ca^2+^, chemotaxis, and secretion of beta-glucuronidase.

Other known NOX2-activators include alkanes C*_n_*H_2_*_n_*_+2_ (*n* = 10–13) and phytol, which are active in the millimolar concentration range (24, 52) as well as a series of patented quinolinone derivatives (53).

### NOX2-Independent ROS Modulators

#### Electron-Deficient Compounds Reactive With Sulfur- and Selen-Containing Biomolecules

A number of electron-deficient (electrophilic) chemical compounds increasing intracellular ROS amount in transformed and proliferating cells have been reported (Figure 3D). However, they are not necessarily applicable for modulation of ROS in neutrophils, which are terminally differentiated cells. For example, arsenic trioxide (As_2_O_3_, Trisenox) is a clinically approved inorganic drug for the treatment of acute promyelocytic leukemia. It is a relatively soft electrophile, which can coordinate soft nucleophilic sulfhydryl and selen-containing biomolecules in cells (54). For example, glutathione (GSH)-depleted cancer cells are especially sensitive to As_2_O_3_ indicating the important role of this tripeptide in As_2_O_3_-detoxification (55). The sulfhydryl- and selen-containing biomolecules including, e.g., GSH, glutathione reductase, and thioredoxin reductase participate in neutralization of ROS in the cells. Their deactivation by As_2_O_3_ usually leads to ROS increase in mammalian cells, which is believed to be one of the reasons of the anticancer activity of this drug. However, though the treatment of neutrophils with As_2_O_3_ causes their apoptosis, the latter is not associated with increase of the intracellular ROS amount (56). One possible explanation of this fact is the higher antioxidant capacity in neutrophils than that of As_2_O_3_^–^ sensitive cancer cells. In particular, it has been found that even during the oxidative burst the amount of bulk antioxidants in neutrophils including GSH/GSSG, ascorbate, and vitamin E is not significantly altered (57).

Some highly potent drugs affecting homeostasis of intracellular thiols were found to induce oxidative stress also in myeloid cells. For example, l-buthionine sulfoximine (BSO, Figure 3D), an inhibitor of gamma-glutamyl-cysteine-synthase (γGCS) and suppressor of GSH synthesis, was found to enhance the oxidative stress in neutrophils and in cultured myeloid progenitors (58). This effect was also reproduced *in vivo* both for wild-type and X-linked CGD mice (59). Since NOX2 is not functional in the CGD mice, the BSO-induced ROS increase in the latter case should be NOX2 independent. In general, the effect of BSO and other GSH modulators on ROS production in neutrophils is substantially weaker than that in cancer cells. One possible explanation of that is the low level of GSH-dependent antioxidant enzymes in neutrophils, whose antioxidative protection seems to rely more strongly on the catalase activity (60).

#### Modulators of the Intracellular Concentration of Ca^2+^ ions

Ca^2+^ ions are present in high concentrations in extracellular space and in the intracellular organelle ER. Release of this metal ion into the cytoplasm triggers a number of biochemical processes including, e.g., NET-formation and mitochondrial permeability transition (61–63). Ca^2+^ transfer across the membrane can be induced by Ca^2+^ ionophores, e.g., A23187 or ionomycin. The ROS-enhancing effects of these ionophores were demonstrated for neutrophils as well as other cells (61–63). However, ROS produced by NOX2 are not essential for the effects of both ionophores, e.g., on formation of NETs (61).

#### Inositol Mimics

γ-Hexaclorocyclohexane (common abbreviation HCCH, other name Lindane) is a potent insecticide. It is also used for the treatment of scabies and lice infestation. However, these pharmaceutical applications have been restricted in many western countries due to pronounced side effects of Lindane, including neuro-, nephro-, and hepatotoxicity. Moreover, this compound was classified as carcinogenic for humans (group 1). Since Lindane is structurally related to inositol, it affects the phosphatidylinositol (PI) cycle in many cells (64), including those of myeloid origin. In particular, this drug triggers ROS amplification in pulmonary alveolar macrophages that is accompanied by increase of the PI turnover and intracellular Ca^2+^ concentration (65). The modulation of ROS and intracellular Ca^2+^ by Lindane was also observed in neutrophils. Additionally, this reagent was found to stimulate degranulation, but did not act as a chemotactic agent, which distinguishes it from stimulants like PMA (66). Apart from being an inositol mimic, HCCH is a hydrophobic molecule. It seems to be aggregated in aqueous solution as evidenced by its concentration-dependent octanol-water partition coefficients ranging from logP = 3.7 at 10 mg/L to logP = 3.9 at <0.1 μg/L (67). Due to these hydrophobic properties, Lindane can interact efficiently with cellular membranes leading to reorganization of phospholipids. In such altered membranes the phospholipids are more exposed to the external factors and, therefore, prone to degradation (68). One of the products formed during this degradation process is arachidonic acid (AA). AA can stimulate ROS production at least *via* two alternative pathways. In particular, AA as well as its metabolites can potentially activate NOX2 (69). Moreover, AA inhibits complex I and III of the mitochondrial respiratory chain that causes electron leakage and generation of superoxide anion radicals as well as other ROS (70). Since NOX2 inhibitors do not attenuate the HCCH-induced ROS-modulation in neutrophils, one can conclude that the mitochondrial AA-mediated pathway predominates in the Lindane-induced oxidative stress in the cells of this type.

#### Effectors of Mitochondrial ROS

Thioglitazones, e.g., pioglitazone and related derivatives, are drugs exhibiting insulin-sensitizing properties (71). They are approved for the treatment of type 2 diabetes. Moreover, these compounds are known to have moderate antiproliferative and anti-inflammatory activity (72). The mechanism of action of thioglitazones relies on activation (agonist properties) of peroxisome proliferator-activated receptor γ, which is a transcription factor of the nuclear receptor family. Moreover, they are known to induce production of mitochondrial ROS both in neutrophils and other cell types (73, 74) due to inactivation of complex I of the mitochondrial respiratory chain (75). Other common inhibitors of the complex I including rotenone (a natural compound, used as insecticide and pesticide) and metformin (a synthetic compound, first-line drug for the treatment of diabetes type 2) also induce ROS generation in neutrophils (76). As expected, this effect is not NOX2-dependent. Therefore, these drugs can be used for ROS modulation in NOX2-deficient states, as it has been demonstrated for pioglitazone in gp91^phox−/−^ mice (a CGD model) and *ex vivo* for primary cells from X-linked CGD patients (74).

### Neutrophil-Specific ROS Modulation

It has been convincingly confirmed *in vitro* and *in vivo* in several animal models of human inflammatory and autoimmune diseases that controlled increase of ROS can be beneficial for the pathological conditions caused by insufficient NOX2 activity in neutrophils, e.g., CGD (74), RA (7, 24), and systemic lupus erythematosus (77). Therefore, it would be appealing to use ROS amplifiers for the treatment of these and related diseases. However, known drugs of this type exhibit a number of undesired side effects due to their influence on other cells than neutrophils. Therefore, for the further clinical development of ROS-enhancing therapies, drugs specific toward neutrophils and able to trigger ROS production at the desired time points, including, e.g., pathogen challenge in CGD, are desirable.

The targeting of the therapeutic agents can in principle be conducted by using approaches developed for non-invasive tracking of neutrophils *in vivo*. For example, neutrophils were successfully labeled and monitored using quantum dots conjugated to a monoclonal antibody, raised against GPI-anchored myeloid differentiation marker (Ly6G or Gr1).

Though the labeled neutrophils remained fully functional in this case (78), higher loading of the drug would be necessary for a functional therapy approach. At these conditions antibodies against Ly6G (per example clone 1A8) are known to cause neutropenia *in vivo* (79). Another known approach for neutrophil labeling in mice makes use of targeting FPR. In particular, it has been demonstrated that conjugates of non-natural peptide ligand cinnamoyl-F-(D)L-F-(D)-L-FK (cFLFLF) with either the radioactive ^64^Cu complex (80) or the near-infrared fluorescence imaging probe Cy7 (81) can be used for neutrophil labeling and monitoring in mice: binding affinity of the Cu-conjugate to FPR was found to be ~18 nM. The weak agonistic effect of cFLFLF on FPR is not crucial in this case, since low loading of the detectable moiety is sufficient for the labeling. However, higher loading, required for functional therapy, is expected to cause unspecific neutrophil activation triggering an unwanted immune response.

Albertine and Gee (82) have reported on an alternative, receptor-independent approach for neutrophil labeling *in vivo*. In particular, they applied the hydrophobic cationic dye PKH26, which is commercially available from Merck (previously Sigma-Aldrich). Under optimized conditions this dye forms micro-aggregates, which are preferentially taken up by neutrophils *in vivo*. However, it has been found later on that these aggregates are also taken by other phagocytic cells, e.g., macrophages. Though at the concentrations required for labeling PKH26 is not toxic, in the presence of day light the toxicity is dramatically increased (83). Furthermore, PKH26 and related structures containing a positively charged polar head and two hydrophobic tail groups at higher concentrations, which would be required for the therapy, can potentially induce lysis of red blood cells. Thus, we conclude that all known neutrophil-targeting approaches are not ideal for therapeutic applications.

**Figure 4:**
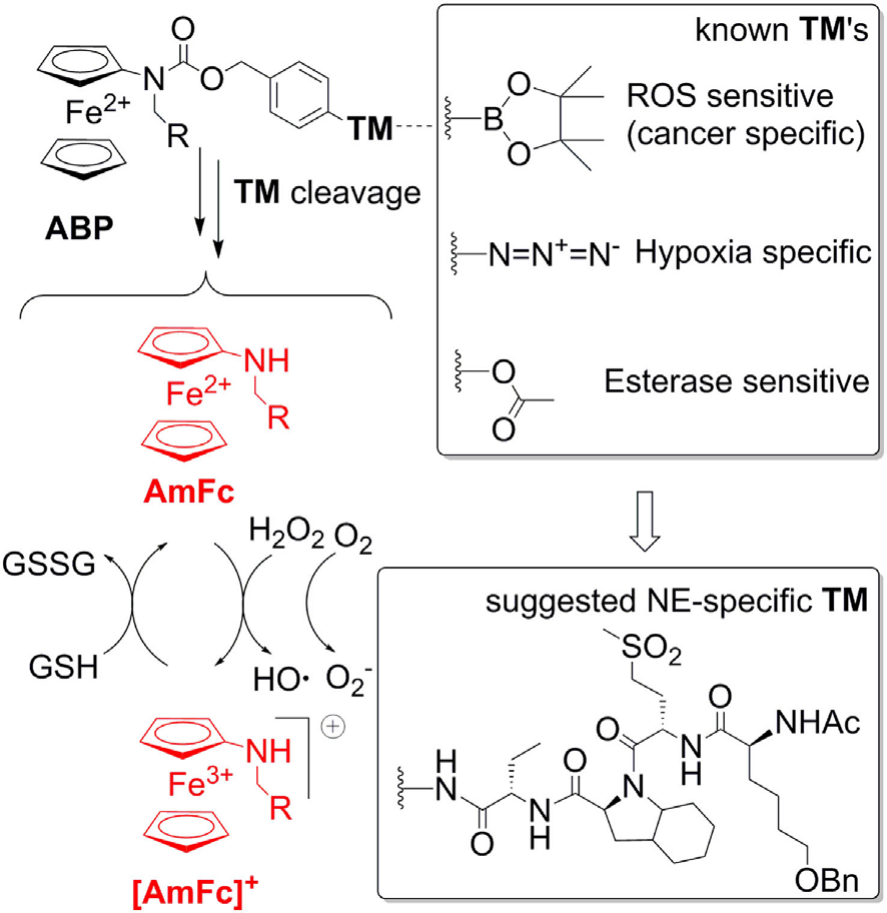
A general structure of aminoferrocene-based prodrugs (ABPs) and the mechanisms of their activation after cleavage of the triggering moiety (TM). The NE-sepcific TM is based on the peptide reported in Ref. (92).

This warrants further research efforts in this field. A possible solution of the problems addressed above is the use of aminoferrocene-based prodrugs (ABP), which were originally developed for targeting cancer cells (84–91). A structure of the parent ABP is shown in Figure 4. Here the ferrocene fragment is a functional unit, which is covalently attached to an electron-acceptor protecting group (arylmethyloxycarbonylamino). Therefore, the molecules of this type are relatively electron-deficient and, correspondingly, do not act as electron donors both in the extracellular space and in cells (84). Furthermore, the ABP contains a triggering moiety (TM). Its removal leads to formation of unstable phenol or aniline derivatives, which spontaneously undergo 1,6-elimination with formation of substituted aminoferrocenes (AmFc). The AmFc derivatives are substantially more electron-rich than the parent ABPs (the redox potential ΔE_1/2_ is shifted by ca. 0.3V). Therefore, in contrast to the ABPs, they act as electron donors for endogenous H_2_O_2_ and ^3^O_2_ leading to formation of highly reactive ROS (HO• and ${\text{O}}_{2}^{-}•$) as well as ferrocenium cations [AmFc]^+^. The latter product can be reduced back to the AmFc by endogenous bulk reducing agents (GSH, ascorbate, and NADPH), thereby closing the catalytic cycle. The ABPs were found to be powerful ROS amplifiers both *in vitro* (experiments with cell cultures and primary cells) and *in vivo* (experiments with mice and rats). By the variation of the TM moiety one can potentially design any cell-specific prodrug providing the cell-specific enzymatic activity is known. For example, already validated TM’s include aryl boronic acid esters (cancer cell-specific and activated under conditions of enhanced oxidative stress), arylazides (hypoxia-specific activation), and carboxylic acid ester (activation in the presence of esterases). Therefore, it is of high interest to explore the possibility of using these versatile prodrugs for design of neutrophil-specific ROS amplifiers.

### Current Ideas and Future Perspectives

In pilot studies, we confirmed that a representative ABP compound, MIS43 (81), is able to enhance ROS in normal neutrophils. This effect is not inhibited by DPI, which indicates that it is not NOX2-dependent. We observed that at the selected experimental conditions MIS43 is a more potent ROS amplifier than known ones including Lindane and BSO, whereas the effect of PMA was found to be stronger (unpublished results).

To create a neutrophil-specific ROS amplifier, we suggest incorporating neutrophil elastase (NE)-specific moieties as TMs in the ABP structure (Figure 4). We selected NE as a neutrophil target, since it is released in the extracellular space and in phagosome and activated only upon neutrophil stimulation, whereas its activity is absent in inactive neutrophils. Moreover, it is known that NOX2-deficient neutrophils release large amounts of NE (93). Therefore, potentially such NE-specific ABPs can be used to enhance ROS specifically in NOX2-deficient neutrophils, which are relevant for CGD, RA, and lupus. As a NE-specific TM one can select a reactive fragment from the number of reported NE-specific fluorogenic substrates. For example, the non-natural tetrapeptide Ac-Nle(OBzl)-Met(O)_2_-Oic-Abu- could be suitable. In particular, this tetrapeptide is NE-specific. Moreover, since it is an artificial structure, it is not expected to be unspecifically degraded *in vivo* (92).

## Author Contributions

VR helped in drafting the manuscript and the figures. JH, CM, CC, LM, MaHe, and MaHo provided scientific input to the manuscript. AM provided the first draft and finalized the manuscript.

## Conflict of Interest Statement

The authors declare that the research was conducted in the absence of any commercial or financial relationships that could be construed as a potential conflict of interest.
